# Oropharyngeal Packing in Nasal Surgery: Effects on Gastric Fullness and Perioperative Safety

**DOI:** 10.4274/TJAR.2026.262390

**Published:** 2026-06-26

**Authors:** Serkan Telli, Süleyman Camgöz, Merve Yaman

**Affiliations:** 1 Kütahya Health Sciences University Department of Anaesthesiology and Reanimation, Kütahya, Türkiye

**Keywords:** Nasal surgical procedures, gastric ultrasonography, oropharyngeal packing, postoperative nausea and vomiting, perioperative care

## Abstract

**Objective:**

To investigate whether drinking-water-moistened oropharyngeal packing during nasal surgery is associated with ultrasound-assessed gastric fullness and postoperative nausea and vomiting (PONV), compared with no packing.

**Methods:**

This single-center, retrospective before-after cohort study included 118 adults undergoing nasal surgery, following an institutional protocol change on December 1, 2024. Sixty patients received oropharyngeal packing moistened with drinking water, and 58 received no packing. All patients received standardized anaesthesia and PONV prophylaxis with dexamethasone 4 mg IV administered after intubation. Packing was placed before surgery and removed before extubation. PONV and throat-related symptoms were recorded at 30 minutes, 2 hours, and 24 hours postoperatively. Gastric fullness was assessed ultrasonographically by measuring gastric antral cross-sectional area (GCSA) and gastric volume before and after extubation.

**Results:**

At 30 minutes, PONV was more frequent in the no-packing group (58.6% vs. 18.3%, *P* < 0.001), as was sore throat (41.4% vs. 26.7%, *P*=0.035). At 2 hours, PONV remained more frequent in the no-packing group (31.0% vs. 13.3%, *P*=0.026), but there was no difference at 24 hours. GCSA and gastric volume decreased in the packing group but increased in the no-packing group (GCSA: -14.14% vs. 20.86%; gastric volume: -15.67% vs. 22.00%; both *P* < 0.001). Demographics, surgical variables, dysphagia, hoarseness, and analgesic/rescue antiemetic use were similar.

**Conclusion:**

Drinking-water-moistened oropharyngeal packing was associated with lower postoperative gastric fullness and a lower incidence of early PONV, without increased throat-related symptoms or rescue medication use. These findings indicate associations, not causal effects, and require confirmation in randomized trials.

**Main Points**

• Drinking-water-moistened oropharyngeal packing during nasal surgery was associated with a lower incidence of early postoperative nausea and vomiting compared with no packing.

• Gastric ultrasonography enabled an objective bedside assessment of postoperative gastric fullness using the gastric antral cross-sectional area and estimated gastric volume.

• Postoperative throat-related symptoms were assessed as safety outcomes, which support the technique moistened with drinking water compared with no oropharyngeal packing.

## Introduction

Nasal procedures, including septoplasty, rhinoplasty, and functional endoscopic sinus surgery (FESS), are frequently performed.^1^ Because the nasal cavity and paranasal sinuses are highly vascular, perioperative bleeding and irrigating fluids may be swallowed and thereby reach the stomach.^2^ Blood ingestion is a recognized contributor to postoperative nausea and vomiting (PONV) in this setting, and published series report a wide range of PONV rates after nasal surgery (34-60%).^3, 4^

PONV can negatively affect patient comfort, delay recovery, and lead to serious complications, including electrolyte imbalance, epistaxis, and aspiration. It also increases healthcare costs by prolonging postoperative stays and elevating readmission rates.^2, 5^ Therefore, effective prevention and management of PONV remain a priority in current anaesthesia practice.^6, 7^

Strategies to prevent PONV include pharmacological and non-pharmacological approaches. Pharmacological prophylaxis typically involves the combined administration of agents with different mechanisms of action, such as antagonists of 5-hydroxytryptamine type 3, dopamine D_2_, histamine H_1_, muscarinic cholinergic, and neurokinin-1 receptors, and corticosteroids, with multi-agent protocols recommended based on individual patient risk.^5, 7^ However, pharmacological interventions alone are often insufficient; non-pharmacological approaches that limit the swallowing of blood, particularly in nasal surgery, continue to play an important role.

Oropharyngeal packing is one of the most commonly used non-pharmacological interventions for managing PONV. Oropharyngeal packing, by creating a physical barrier in the oropharynx, is intended to reduce the passage of blood and irrigation fluids into the stomach, thereby potentially limiting gastric volume expansion and reducing the risk of PONV.^2, 3, 8, 9^ Despite these potential benefits, oropharyngeal packing is not without its complications. Dry oropharyngeal packing is associated with postoperative sore throat, edema, and mucosal ulceration, which result from compression and dryness.^2^ The present study evaluated oropharyngeal packing moistened with drinking water versus no oropharyngeal packing.

The effects of oropharyngeal packing on PONV are inconsistent in the literature. While Jin et al.^2^ reported that oropharyngeal packing did not significantly reduce PONV, a recent randomized controlled trial by Altun et al.^9^ demonstrated that oropharyngeal packing significantly reduced the gastric antral cross-sectional area (GCSA). However, the method of moistening the pack (physiological saline, drinking water, or other solutions) and its effect on mucosal irritation have not been sufficiently clarified.

Gastric ultrasonography is a non-invasive method for assessing gastric fullness.^10^ By measuring the cross-sectional area of the gastric antrum, ultrasonography provides an objective estimate of gastric volume and helps contextualize aspiration risk. Accordingly, changes in gastric volume are appropriate parameters for evaluating the association between oropharyngeal packing and gastric fullness. In this study, we examined the association of drinking-water-moistened oropharyngeal packing with PONV (primary outcome, assessed within 24 hours), ultrasound-derived gastric fullness metrics, and throat-related symptoms (secondary outcomes).

## Methods

This single-center, retrospective, before-after cohort study, conducted at a tertiary care center, was based on an institutional protocol change implemented on December 1, 2024, whereby drinking-water-moistened oropharyngeal packing replaced no packing as routine practice during nasal surgery. Patients who underwent surgery before this date constituted the no-packing (control) group, and those who underwent surgery on or after this date constituted the packing group. No other changes were made to the perioperative protocols, including anaesthesia induction and maintenance, antiemetic prophylaxis, or postoperative analgesia. Ethical approval was obtained from the Kütahya University of Health Sciences Non-Interventional Clinical Research Ethics Committee (approval no.: 2025/08-50, date: 19.06.2025). The requirement for informed consent was waived due to the retrospective nature of the study. The study was conducted in accordance with the Declaration of Helsinki and was reported according to the STROBE guidelines.

Records of patients who underwent septoplasty, rhinoplasty, or FESS between April 1, 2024, and April 15, 2025, were reviewed. Patients aged 18-67 years who were classified as American Society of Anesthesiologists (ASA) physical status I-II, had a body mass index (BMI) <35 kg×m^-2^, and underwent the standardized anaesthesia protocol were included. The exclusion criteria were pregnancy, active smoking, uncontrolled diabetes mellitus, a history of gastroesophageal reflux disease, and incomplete records. The final study population consisted of 118 patients: 60 in the packing group and 58 in the no-packing group.

### Anaesthesia and Packing Protocol

All patients received the same standardized anaesthesia protocol. Premedication consisted of intravenous midazolam (0.03 mg kg^-1^). Anaesthesia was induced with fentanyl (1 µg kg^-1^), propofol (2 mg kg^-1^), and rocuronium (0.6 mg kg^-1^). Immediately after endotracheal intubation, all patients received intravenous dexamethasone 4 mg as standard PONV prophylaxis, in accordance with institutional practice; no additional prophylactic antiemetics were administered. Anaesthesia was maintained with 2% sevoflurane in a 50% O_2_/air mixture using a cuffed endotracheal tube (7.5-8.0 mm internal diameter). At the end of the surgery, neuromuscular blockade was reversed, and the patients were extubated. Postoperative analgesia consisted of intravenous administration of paracetamol (1 g) and tramadol hydrochloride (100 mg).

In the packing group, oropharyngeal packing was performed under sterile conditions using sterile gauze pads moistened with drinking water at room temperature. With the patient supine and the head in a neutral position, the pack was placed in the oropharynx without excessive pressure before the start of surgery and was removed before extubation. All intraoperative procedures were performed by experienced anaesthesiologists following a standardized institutional protocol.

### Gastric Ultrasonography

Gastric ultrasonography is routinely performed in all surgical patients at our institution as part of the standard perioperative assessment and anaesthesiology resident training; it was not introduced specifically for this study. Gastric ultrasound was performed using an Affiniti 50 system (Philips, USA) equipped with a 4-12 MHz linear probe, while the patient was in the right lateral decubitus position. The gastric antrum was identified in the sagittal view using adjacent anatomical landmarks (left liver lobe, abdominal aorta, and superior mesenteric artery). The anteroposterior (D1) and craniocaudal (D2) antral diameters were measured serosa-to-serosa, and the GCSA was calculated using the standard ellipse-based approach (GCSA=π×D1×D2/4). GCSA was recorded preoperatively and 30 minutes after extubation by a single experienced anaesthesiologist who was not involved in the intraoperative care. This approach ensured measurement consistency but precluded the assessment of inter-observer reliability. The estimated gastric volume was derived from the Perlas model [mL=27.0+14.6×GCSA (cm^2^)-1.28×age] and expressed as mL kg^-1^.^11, 12^ The percentage change was calculated as follows: (postoperative-preoperative)/preoperative)×100. Due to the non-normal distribution of these variables, the results are reported as the median with interquartile range (IQR).

### Data Collection and Outcome Definitions

Data were retrospectively extracted from the anaesthesia records, postoperative nursing charts, and physician notes. The following parameters were collected: demographics (age, sex, BMI, ASA classification); surgery type and duration; early post-extubation findings (blood in the endotracheal tube, nausea, vomiting, gag reflex, cough, hoarseness, laryngospasm, and other complications); PONV, sore throat, dysphagia, and hoarseness assessed at 30 minutes, 2 hours, and 24 hours postoperatively; use of rescue antiemetics and analgesics within 24 hours; preoperative and postoperative GCSA; and patient satisfaction at 24 hours.

PONV was defined as any episode of postoperative nausea or vomiting documented in the medical records at predefined time points (30 minutes, 2 hours, and 24 hours). In our institutional protocol, all patients received identical PONV prophylaxis (dexamethasone 4 mg IV after intubation), and no other prophylactic antiemetic was administered. Rescue antiemetic medication was administered exclusively to patients who developed postoperative nausea or vomiting. Accordingly, rescue antiemetic administration served as a corroborating marker of symptomatic PONV rather than an independent diagnostic criterion, and no patient was classified as having PONV based on rescue antiemetic use alone. This definition was consistently applied across all postoperative time points.

Patient satisfaction was assessed 24 hours postoperatively using a simple ordinal Likert scale documented in routine clinical records. Patients selected the option that best represented their level of satisfaction: “good,” “very good,” “moderate,” or “poor”. Because this assessment was part of routine clinical documentation, a formally validated satisfaction questionnaire was not prospectively applied, and the responses were recorded as ordinal categories and analyzed accordingly.

### Sample Size

The sample size was calculated using G^*^Power (v3.1), based on the postoperative GCSA. Using the values reported by Altun et al.^9^ (control: 999.4±202.1 mm^2^; packing: 701.1±82.2 mm^2^), Cohen’s d was 1.93. A two-sided t-test with α=0.01 and power (1-β)=0.90 required 12 patients per group; therefore, the 118 patients in the present study provided >99% statistical power. A post-hoc power analysis for the 30-minute PONV incidence using Fisher’s exact test, based on the observed group proportions and the actual sample size, suggested high achieved power (99.8%); however, post-hoc power analyses do not substitute for prospective sample size planning and should be interpreted cautiously.

### Statistical Analysis

Statistical analyses were performed using SPSS version 21.0 (IBM, Armonk, NY, USA). Distributional assumptions were assessed using the Shapiro-Wilk test. Continuous data are summarized as mean (SD) or median (IQR), as appropriate; categorical data are summarized as n (%). Between-group comparisons used the χ^2^ test or Fisher’s exact test for categorical variables, and the Independent Samples t-test or the Mann-Whitney U test for continuous variables. Within-group pre/post comparisons were performed using the Wilcoxon signed-rank test. A two-sided* P* value <0.05 was considered statistically significant.

Multivariable logistic regression analysis was performed to evaluate the association between oropharyngeal packing and PONV at the 30-minute and 2-hour time points, for which event frequency was sufficient. Age, sex, BMI, and group (packing vs. no-packing) were entered as independent variables. Multicollinearity was assessed using the variance inflation factor (VIF) and tolerance statistics (acceptance thresholds: VIF <5, tolerance >0.1). Model fit was assessed using the Hosmer-Lemeshow test, and overall model significance was assessed using the Omnibus test of model coefficients. Adjusted odds ratios (ORs) with 95% confidence intervals (CIs) are reported. At the 24-hour time point, regression analysis was not performed because event frequency was very low. Analysis of covariance (ANCOVA) was used to compare postoperative GCSA and gastric volume between groups, with oropharyngeal packing as the fixed factor and the respective preoperative values entered as covariates. Adjusted means, 95% CI, and effect sizes (partial η^2^) are reported.

## Results

### Baseline Characteristics

A total of 118 patients were analyzed: 60 in the packing group and 58 in the no-packing group. Age, sex, height, weight, BMI, and surgical time were comparable between the groups (all *P* > 0.05; Table 1). The median age was 28 years (range, 18-67) in the packing group and 25.5 years (range, 18-66) in the no-packing group. The median surgical duration was 152.5 minutes in the packing group and 135 minutes in the no-packing group. Septoplasty was the most frequently performed procedure in both groups (packing group: 25%; no-packing group: 31%), and the distribution of FESS, submucosal resection, and other nasal procedures between groups was similar (*P*=0.391).

### Early Post-extubation Findings

The early post-extubation parameters are summarized in Table 2. Nausea was significantly more frequent in the no-packing group than in the packing group (20.7% vs. 5.0%, *P *< 0.01), whereas vomiting frequency did not differ significantly between groups (*P*=0.717). Although the presence of blood in the endotracheal tube did not reach statistical significance (28.3% vs. 39.7%, *P*=0.133), its frequency was lower in the packing group, suggesting a possible trend toward reduced blood contamination. The incidence of cough, hoarseness, laryngospasm, and other adverse events was similar between the groups.

### Postoperative Symptoms at 30 Minutes, 2 Hours, and 24 Hours

Sore throat, PONV, dysphagia, and hoarseness are presented at the three postoperative time points in Table 3. No significant differences were observed between the groups in terms of dysphagia or hoarseness at any time point (all *P* > 0.05). At 30 minutes, sore throat occurred significantly less frequently in the packing group than in the no-packing group (26.7% vs. 41.4%, *P*=0.035). PONV was significantly less frequent in the packing group at 30 minutes (18.3% vs. 58.6%, *P *< 0.001) and at 2 hours (13.3% vs. 31.0%, *P*=0.026); however, the difference was no longer apparent at 24 hours (*P *> 0.05). Nausea-vomiting NRS scores at 30 minutes were also significantly lower in the packing group (0.97±1.15 vs. 4.65±1.73; Z=-8.44, *P *< 0.001; effect size r=0.81). No significant differences were observed between the groups in terms of rescue antiemetic or analgesic requirements during the first 24 hours (*P *> 0.05).

### Multivariable Analysis of PONV

Multivariable logistic regression, adjusted for age, sex, and BMI, was performed for the 30-minute and 2-hour time points. At 30 minutes, 45 patients presented with PONV (11 in the packing group and 34 in the no-packing group); the model was statistically significant [χ^2^(4)=27.551, *P *< 0.001; Nagelkerke R^2^=0.301; Hosmer-Lemeshow *P*=0.671]. Oropharyngeal packing was independently associated with lower odds of PONV (OR: 0.12, 95% CI: 0.05-0.28, *P *< 0.001). At 2 hours, 26 patients had PONV (8 vs. 18). The model was also significant [χ^2^(4)=9.693, *P*=0.046; Hosmer-Lemeshow *P*=0.469], and oropharyngeal packing remained independently associated with lower odds of PONV (OR: 0.32, 95% CI: 0.12-0.84, *P*=0.021). No significant independent association was found between age, sex, BMI, and PONV (all *P *> 0.05). At 24 hours, only one PONV event was observed in the entire cohort; therefore, multivariable regression was not performed. The unadjusted comparison showed no between-group differences (*P*=1.000). The magnitude of the adjusted OR should be interpreted in the context of the retrospective before-after design, in which residual confounding may influence effect size estimates.

### Patient Satisfaction

As an exploratory outcome, distributions of patient satisfaction differed between groups at 24 hours (*P *< 0.001). In the packing group, 70.7% reported “good,” 13.8% “very good,” and 15.5% “moderate,” with no “poor” responses. In the no-packing group, 80.4% reported “moderate,” 13.7% “poor,” 5.9% “good,” and none reported “very good”. As the assessment used a simple non-validated ordinal scale documented in routine charts, these data are only hypothesis-generating.

### Gastric Ultrasonography Findings

The preoperative and postoperative GCSA and gastric volumes are presented in Tables 4 and 5, respectively. Within-group analysis showed a significant postoperative decrease in GCSA and gastric volume in the packing group, whereas these parameters increased significantly in the no-packing group. Between-group comparisons showed that the median percentage change in GCSA was -14.14% in the packing group and +20.86% in the no-packing group, and that the median percentage change in gastric volume was -15.67% in the packing group and +22.00% in the no-packing group (both *P *< 0.001).

ANCOVA was performed to evaluate the independent association of oropharyngeal packing with postoperative GCSA and gastric volume, adjusting for the respective preoperative values. The assumption of homogeneity of variance was satisfied for both outcomes [Levene’s tests: F (1,116)=0.456, *P*=0.501 for GCSA; F (1,116)=0.025, *P*=0.875 for gastric volume]. Preoperative values were significant covariates for postoperative GCSA F (1,115)=47.683, *P *< 0.001, partial η^2^=0.310) and postoperative gastric volume F (1,115)=84.158, *P *< 0.001, partial η^2^=0.443). The adjusted postoperative mean GCSA was lower in the packing group than in the no-packing group (4.39 vs. 6.23 cm^2^; adjusted mean difference -1.84, 95% CI: -2.44 to -1.24; F (1,115)=36.663, *P *< 0.001, partial η^2^=0.257). Similarly, the adjusted mean postoperative gastric volume was lower in the packing group [50.22 vs. 80.12 mL; adjusted mean difference -29.90, 95% CI: -39.06 to -20.74; F (1,115)=41.875, *P *< 0.001, partial η²=0.283]. The overall models explained 38.9% of the variance in postoperative GCSA (adjusted R^2^=0.378) and 50.4% of the variance in postoperative gastric volume (adjusted R^2^=0.495). These findings are consistent with the independent association between oropharyngeal packing and lower postoperative gastric fullness, possibly reflecting reduced ingestion of blood and irrigation fluids during surgery. A lower gastric volume may theoretically be associated with a reduced aspiration risk; however, aspiration events were not directly evaluated in this study, and this link should therefore be regarded as hypothetical.

## Discussion

This retrospective before-and-after cohort study evaluated the association between oropharyngeal packing moistened with drinking water and perioperative outcomes in patients who underwent nasal surgery. The baseline characteristics were comparable between the groups. The packing group had lower rates of early postoperative nausea, vomiting, and sore throat, which were accompanied by objective reductions in GCSA and estimated gastric volume, consistent with possible attenuation of perioperative blood ingestion. Both groups received identical standard PONV prophylaxis (dexamethasone 4 mg IV after intubation), which reduces but does not eliminate the likelihood that the observed differences in PONV were attributable to differential antiemetic management.

Previous studies have reported that conventional (dry) oropharyngeal packs may increase postoperative throat discomfort by causing mucosal compression, friction, and drying.^2, 3, 13, 14^ Evidence regarding moistening techniques is limited; however, some studies suggest that pack hydration may improve mucosal tolerability.^3, 9, 15^ In our cohort, early postoperative sore throat was less frequent among patients who received oropharyngeal packing moistened with drinking water. This observation is consistent with reports suggesting improved comfort when moisture reduces frictional injury. However, because our study did not include a saline-moistened comparison arm, the specific contribution of moistening with water remains uncertain.

The role of oropharyngeal packing in preventing PONV remains controversial. Multiple randomized trials and meta-analyses have reported that oropharyngeal packing, particularly when dry or saline-moistened, does not consistently reduce PONV and may exacerbate early postoperative throat discomfort.^2, 16^ Conversely, other studies have shown reductions in antral cross-sectional area and postoperative gastric volume in patients undergoing packing, suggesting that decreased blood ingestion may mediate these effects.^3, 9^ Meta-analytic findings indicate that the timing of evaluation may contribute to these discrepancies: some studies report higher early PONV in patients with packing, whereas others report higher late PONV in patients without packing.^16^ Furthermore, gastric blood is frequently detected regardless of packing, with variability attributable to material, moisture, and placement technique.^15, 17^

Within this context, the lower early PONV rates and reduced postoperative gastric volumes observed in the packing group align with the findings of studies supporting the role of packing in limiting intraoperative blood ingestion. The disappearance of between-group differences in PONV at 24 hours underscores the multifactorial and time-dependent nature of PONV after nasal surgery. ANCOVA confirmed that postoperative GCSA and gastric volume were lower in the packing group after adjustment for preoperative values. Although the adjusted OR for early PONV was substantial, its magnitude warrants particular caution. The adjusted OR at 30 minutes of 0.12 (95% CI: 0.05-0.28) is unusually large in magnitude for a retrospective before-after design. This estimate likely overestimates the true effect, reflecting inherent design limitations and residual confounding from unmeasured variables, both of which multivariable adjustment cannot fully address. It should therefore be interpreted as an association rather than a causal effect, and the magnitude itself should not be taken at face value.

Although not statistically significant, The trend toward reduced visible blood contamination of endotracheal tubes in the packing group may reflect lower airway exposure. No increase in dysphagia or hoarseness was observed, indicating that the moistened technique did not appear to introduce an additional mucosal burden-a finding consistent with prior reports describing variable tolerability across packing materials and hydration techniques.

Gastric ultrasonography is a useful, non-invasive bedside tool for estimating gastric fullness.^10^ In our study, consistent with previous reports,^3, 9^ postoperative gastric volume increased in patients without packing, whereas moistened oropharyngeal packing was associated with measurable reduction. These objective findings support the plausibility of a packing-related reduction in perioperative blood ingestion, although the observational design precludes causal conclusions.

Patient satisfaction scores were higher in the packing group, possibly reflecting reduced early symptom burden. However, as this was an exploratory finding based on a non-validated ordinal scale, it should not be interpreted as a primary outcome and should be evaluated prospectively with a validated instrument.

### Study Limitations

This study has several limitations inherent in its single-center, retrospective, before-after cohort design. Because group allocation was based on a time-dependent institutional protocol change rather than randomization, temporal confounding cannot be fully excluded. Learning curve effects, staffing changes, secular modifications in perioperative care, and seasonal or case-mix variations may have influenced outcomes independently of oropharyngeal packing. The use of a uniform anaesthesia and antiemetic protocol in both groups reduces the likelihood that differential prophylaxis accounts for the observed PONV differences, but does not eliminate the possibility of other unmeasured confounders.

PONV assessment relied on retrospective chart review rather than on a prospectively applied, validated scale, which may have resulted in underreporting or misclassification of mild symptoms. Similarly, patient satisfaction was evaluated using a simple, non-validated ordinal Likert scale documented in routine charts rather than with a validated questionnaire; thus, these results should be interpreted with caution.

All gastric ultrasound measurements were performed by a single, experienced anaesthesiologist who was not involved in the intraoperative care. Although a standardized scanning protocol and consistent anatomical landmarks were used, complete blinding to group allocation could not be guaranteed given the retrospective design; additionally, inter-observer reliability could not be assessed because all measurements were obtained by a single operator. The absence of assured blinding represents a meaningful limitation: awareness of group allocation, even with a standardized scanning protocol, may have biased postoperative gastric measurements toward the expected direction (i.e., lower values in the packing group), potentially exaggerating the observed between-group differences in GCSA and gastric volume. This possibility should be considered when interpreting the magnitude of these findings.

Finally, because of the non-randomized design, the observed associations—particularly the high adjusted OR for early PONV—should be regarded as hypothesis-generating rather than as evidence of large causal effects. Prospective randomized multicenter trials are required to confirm these findings.

## Conclusion

In this retrospective before-after cohort study, oropharyngeal packing was associated with lower rates of early postoperative sore throat, lower incidence of early PONV, and lower postoperative gastric volume among patients who underwent nasal surgery. These observations suggest that drinking-water-moistened oropharyngeal packing may serve as a simple, low-cost, non-pharmacological adjunct in this setting. However, given the non-randomized, before-after design and the potential for residual confounding, the present findings should be regarded as associative and hypothesis-generating; causality cannot be established within the current study design. Prospective, randomized, multicenter trials are required to confirm these findings and to determine the optimal packing technique and its role in perioperative management.

## Ethics

**Ethics Committee Approval:** Ethical approval was obtained from the Kütahya University of Health Sciences Non-Interventional Clinical Research Ethics Committee (approval no.: 2025/08-50, date: 19.06.2025).

**Informed Consent:** Retrospective study.

## Figures and Tables

**Table 1. Demographic Characteristics of the Patients:** 

-	**Group 1**	**Group 2**	***P* value**
	**(n = 60)**	**(n = 58)**	
**Age (years), med, (min-max)**	28 (18-67)	25.5 (18-66)	0.287*
**Male sex, n (%)**	46 (76.7)	42 (72.4)	0.674**
**Female sex, n (%)**	14 (23.3)	16 (27.6)	
**Height (cm), mean ± SD**	171.48±9.01	173.79±9.55	0.179***
**Weight (kg), mean ± SD**	72.37±13.94	77.14±14.90	0.075***
**BMI (kg×m^-2^), mean ± SD**	24.58±4.28	25.57±4.90	0.245***
**Duration of surgery (min), med, (min-max)**	152.50 (70-480)	135 (60-385)	0.162*

*: Mann-Whitney U test; **: Chi-square test; ***: Independent Samples t-test Group 1, packing group; Group 2, no-packing group; BMI, body mass index; SD, standard deviation; min-max, minimum-maximum

**Table 2. Initial Clinical Findings in the Early Post-extubation Period:** 

-	**Group 1 (n = 60)**		**Group 2 (n = 58)**		-
	**Present (n, %)**	**Absent (n, %)**	**Present(n, %)**	**Absent (n, %)**	*P* value*
**Blood in the endotracheal tube**	17 (28.3%)	43 (71.7%)	23 (39.7%)	35 (60.3%)	0.133
**Nausea**	3 (5.0%)	57 (95.0%)	12 (20.7%)	46 (79.3%)	**<0.01**
**Vomit**	5 (8.3%)	55 (91.7%)	3 (5.2%)	55 (94.8%)	0.717
**Gagging**	10 (16.7%)	50 (83.3%)	10 (17.2%)	48 (82.8%)	1.000
**Cough**	11 (18.3%)	49 (81.7%)	16 (27.6%)	42 (72.4%)	0.276
**Hoarseness**	2 (3.3%)	58 (96.7%)	0 (0.0%)	58 (100.0%)	0.496
**Laryngospasm**	2 (3.3%)	58 (96.7%)	1 (1.7%)	57 (98.3%)	1.000
**Additional complication**	1 (1.7%)	59 (98.3%)	1 (1.7%)	57 (98.3%)	1.000

Group 1, packing group; Group 2, no-packing group

*: Fisher’s exact test

**Table 3. Postoperative Assessments at 30 Minutes, 2 Hours, and 24 Hours:** 

-		**Group 1 (n = 60)**		**Group 2 (n = 58)**		*P* value*
		**Present (n, %)**	**Absent (n, %)**	**Present (n, %)**	**Absent (n, %)**	
**30 min**	Throat pain	16 (26.7%)	44 (73.3%)	24 (41.4%)	34 (58.6%)	**0.035**
	Nausea/vomit	11 (18.3%)	49 (81.7%)	34 (58.6%)	24 (41.4%)	**<0.001**
	Dysphagia	4 (6.7%)	56 (93.3%)	3 (5.2%)	55 (94.8%)	1.000
	Hoarseness	2 (3.3%)	58 (96.7%)	2 (3.4%)	56 (96.6%)	1.000
**2 hour**	Throat pain	17 (28.3%)	43 (71.7%)	18 (31.0%)	40 (69.0%)	0.841
	Nausea/vomit	8 (13.3%)	52 (86.7%)	18 (31.0%)	40 (69.0%)	**0.026**
	Dysphagia	5 (8.3%)	55 (91.7%)	3 (5.2%)	55 (94.8%)	0.717
	Hoarseness	2 (3.3%)	58 (96.7%)	1 (1.7%)	57 (98.3%)	1.000
**24 hour**	Throat pain	2 (3.3%)	58 (96.7%)	1 (1.7%)	57 (98.3%)	1.000
	Nausea/vomit	1 (1.7%)	59 (98.3%)	0 (0.0%)	58 (100.0%)	1.000
	Dysphagia	0 (0.0%)	60 (100.0%)	0 (0.0%)	58 (100.0%)	1.000
	Hoarseness	2 (3.3%)	58 (96.7%)	0 (0.0%)	58 (100.0%)	0.496

Group 1, packing group; Group 2, no-packing group

*: Fisher’s exact test

**Table 4. Intragroup Comparison of Preoperative and Postoperative GCSA (cm2) and Gastric Volume Measurements (mL):** 

-	**Group 1 (n = 60)**				**Group 2 (n = 58)**			
	**Preop median (IQR)**	**Postop median (IQR)**	**Median difference**	*P* value*	**Preop median (IQR)**	**Postop median (IQR)**	**Median difference**	*P* value*
**GCSA (cm^2^)**	4.82 (2.80)	4.32 (2.19)	-0.50	**<0.001**	4.50 (2.47)	5.80 (2.38)	1.30	**<0.001**
**Gastric Volume (mL)**	60.20 (33.75)	50.61 (42.63)	-9.59	**<0.001**	56.86 (43.08)	71.86 (32.12)	15.00	**<0.001**
**Gastric Volume kg^-1^ (mL kg^-1^)**	0.81 (0.61)	0.66 (0.69)	-0.15	**<0.001**	0.74 (0.53)	1.06 (0.47)	0.32	**<0.001**

Group 1, packing group; Group 2, no-packing group. GCSA, gastric antral cross-sectional area; IQR, interquartile range. *: Wilcoxon signed-rank test

**Table 5. Intergroup Comparison of Preoperative and Postoperative GCSA (cm2) and Gastric Volume Measurements (mL):** 

-	**Group 1 (n = 60)** **median (IQR)**	**Group 2 (n = 58)** **median (IQR)**	**Z value**	*P* value^*^
**Preop GCSA (cm^2^)**	4.82 (2.80)	4.50 (2.47)	-2.201	**0.028**
**Postop GCSA (cm^2^)**	4.32 (2.19)	5.80 (2.38)	-3.662	**<0.001**
**Preop gastric volume (mL)**	60.20 (33.75)	56.86 (43.08)	-1.251	0.211
**Postop gastric volume (mL)**	50.61 (42.63)	71.86 (32.12)	-3.416	**0.001**
**Preop gastric volume kg^-1^ (mL kg^-1^)**	0.81 (0.61)	0.74 (0.53)	-1.634	0.101
**Postop gastric volume kg^-1 ^(mL kg^-1^)**	0.66 (0.69)	1.06 (0.47)	-2.815	**<0.01**
**Δ** **GCSA** **(%)**	-14.14 (22.22)	20.86 (44.57)	-7.169	**<0.001**
**Δ** **Gastric volume** **(%)**	-15.67 (32.73)	22.00 (53.84)	-7.173	**<0.001**

Group 1, packing group; Group 2, no-packing group; GCSA, gastric antral cross-sectional area; IQR, interquartile range; ΔGCSA, percentage change in GCSA; ΔGastric volume, percentage change in gastric volume

*: Mann-Whitney U test
